# Radiosurgery to the Medial Thalamus for Chronic Pain: A Single Group Experience and Review of Literature

**DOI:** 10.7759/cureus.83003

**Published:** 2025-04-25

**Authors:** Paola Del Cid, Alejandra Moreira, Fidel Campos, Claudia Cruz, Alejandro Blanco, Eduardo E Lovo

**Affiliations:** 1 Medicine, Dr. José Matías Delgado University, San Salvador, SLV; 2 Neurosurgery, International Cancer Center, San Salvador, SLV; 3 Radiosurgery, International Cancer Center, Diagnostic Hospital, San Salvador, SLV; 4 Anesthesia and Pain Management, Diagnostic Hospital, San Salvador, SLV; 5 Radiosurgery, Robotic Radiosurgery Center, San Jose, CRI; 6 Neurosurgery-Gamma Knife Program, International Cancer Center, Diagnostic Hospital, San Salvador, SLV

**Keywords:** intractable pain, neuropathic pain, oncologic pain, stereotactic radiosurgery, thalamotomy

## Abstract

Introduction

Chronic pain presents a significant challenge in achieving effective treatment and can greatly reduce patients’ quality of life. Many individuals can undergo multiple therapies with limited success. Stereotactic medial thalamotomy is a treatment option for certain types of chronic pain when other measures have failed. This study aims to assess the efficacy and safety of radiosurgical medial thalamotomy based on nearly a decade of experience in managing chronic intractable pain of both malignant and non-malignant origins.

Methods

This retrospective study included 56 patients who underwent radiosurgery to the medial thalamus for refractory neuropathic pain or mixed cancer pain. Treatment approaches involved single thalamic irradiation, bilateral thalamotomy, dual-target irradiation of the medial thalamus and trigeminal nerve, or triple-target irradiation of the pituitary gland and bilateral thalami.

Results

Pain intensity was significantly reduced at the final follow-up for patients treated with triple-target radiosurgery (Student’s t-test, p < 0.001), dual-target radiosurgery (Wilcoxon test, p < 0.001), and single/bilateral thalamotomy (Wilcoxon test, p = 0.01). The median time to pain relief was 2.5 days for triple-target treatment, three days for dual-target treatment, 1.5 days for bilateral thalamotomy, and 33 days for single thalamotomy. Overall, treatment success, defined as achieving at least 50% pain relief, was 69.1% for the entire cohort. The success rate was 66.6% for oncologic pain and 71.7% for non-oncologic pain.

Conclusion

Radiosurgical irradiation of the medial thalamus is an effective alternative for treating refractory cancer-related pain and predominantly neuropathic non-oncologic pain. Various radiosurgical regimens can be employed in a multi-target strategy tailored to the origin and characteristics of the pain, offering relief to most patients with minimal morbidity.

## Introduction

Chronic refractory pain includes a variety of pain syndromes that present significant challenges in treatment and impose substantial human and healthcare costs worldwide [1]. Although various interventions, such as pharmacological, neuromodulatory, surgical, and intrathecal therapies, are available for pain relief, 20% to 30% of patients may still experience refractory pain, leading to considerable socioeconomic and psychosocial burdens [2].

Radiosurgery is an important treatment option for chronic facial pain, particularly trigeminal neuralgia, though guidelines for managing other related conditions remain less clear [3]. Trigeminal neuralgia is characterized by paroxysmal, electric shock-like pain triggered by specific stimuli. However, it may present with more complex features, such as continuous pain, persistent idiopathic facial pain, deafferentation pain, secondary neuralgias, or other types of facial pain that are not of neuropathic origin. These diverse presentations can complicate diagnosis and management, often resulting in polypharmacy and multiple surgical or radiosurgical procedures, which may yield suboptimal outcomes. Even when the diagnosis is certain and treatments such as microvascular decompression, radiosurgery, or other percutaneous procedures are performed, up to 20% of patients may remain refractory to these gold-standard interventions [4].

Pain associated with malignancy presents additional challenges, as 40% of cancer patients experience a combination of nociceptive, somatic, visceral, or neuropathic pain that may be continuous, episodic, or exacerbated by breakthrough crises [5]. This complex interplay of pain mechanisms causes 75% of patients with advanced cancer to become refractory to standard treatments, thereby increasing morbidity and suffering for both patients and caregivers [6]. Additionally, healthcare costs rise due to emergency visits, hospitalizations, and advanced pain management procedures in oncological settings.

Gamma Knife stereotactic radiosurgery (SRS) has been used since its inception for the radiosurgical lesioning of key structures within pain pathways to provide relief when other treatments have failed. Targets such as the medial thalamus and pituitary gland have been used for both malignant and non-malignant pain, with varying degrees of success [3]. Compared to other invasive surgical approaches, these radiosurgical methods generally result in fewer complications. Due to its role in pain perception and its favorable safety profile, the medial thalamus, particularly the centromedian-parafascicular complex, is a well-recognized target for treating intractable pain, either alone or in combination with other targets, as previously described by our group [7].

This study presents nearly a decade of experience with medial thalamic irradiation for intractable malignant and non-malignant pain. We describe the outcomes of different radiosurgical strategies, including irradiation of the thalamus alone, bilaterally, or in combination with the trigeminal nerve, pituitary gland, or cingulum; as well as a review of the current literature regarding these interventions.

## Materials and methods

This retrospective study evaluated 56 patients who underwent irradiation of the medial thalamus for mixed oncological pain or neuropathic pain, most often related to refractory chronic facial pain of various origins. The Centro Internacional de Cancer, Hospital de Diagnostico Ethics Committee locally approved the study (Approval number Ref. CECIC 2024/001). All patients had a history of insufficient pain control in spite of adequate pharmacological, minimally invasive or invasive treatment measures. As such, they were deemed refractory by a specialized team consisting of neurosurgeons, pain management specialists, and palliative care providers (in the case of oncological patients for whom no other treatment options were available or feasible). Diagnostic protocols for non-oncological pain involved evaluations by neurology, neurosurgery, and neuropsychiatry, and a detailed assessment by the center’s protocol selection team before SRS.

Pain was assessed using the Numerical Rating Scale (NRS), which rates pain intensity on a scale of 1 to 10. For patients with chronic facial pain who experienced paroxysmal episodes but were not in a crisis at the time of treatment, the NRS score for the most recent crisis was recorded. For each patient, affected sites, pain triggers, frequency and duration of pain crises (in minutes), baseline and crisis-related medications, and daily activities impeded by pain were documented. Additionally, the EuroQol-5 Dimension (EQ-5D) assessed quality of life, and trigeminal neuralgia patients were evaluated using the Barrow Neurological Index (BNI).

Follow-up was conducted via telemedicine according to patient and caregiver preferences. Self-reported data were collected most frequently. Follow-up was daily during the first week, once at two weeks, and then monthly for up to 12 months, or until the patient died in cases of oncological pain. After 12 months, annual follow-up was provided when possible. NRS, BNI, and EQ-5D scores were recorded at each follow-up, along with any changes in medication regimens or radiation-related side effects. Patients were advised to continue their pain medications as directed by their primary care physician.

Statistical analysis

All statistical analyses were performed to evaluate changes in pain intensity and treatment outcomes. Pain intensity, measured using the NRS, was compared pre- and post-treatment. Differences in NRS scores were assessed using a Student’s t-test for patients with normally distributed data, such as patients in the triple-target and single/bilateral thalamotomy groups. The Wilcoxon signed-rank test was used for comparisons in patients with non-normally distributed data, such as those who received dual-target radiosurgery. A p-value of less than 0.05 was considered statistically significant. Overall treatment success was defined as a reduction of at least 50% in NRS scores; treatment responses were further stratified into complete relief (100% reduction of initial NRS scores), good (50 to 99%), poor (1 to 49% NRS reduction), or none (0% NRS reduction). Analyses also included recurrence rates of severe pain and comparisons of response times among different treatment groups.

## Results

Patient cohort

Between 2016 and 2024, our group treated 56 patients for intractable pain of malignant or non-malignant origin. Radiosurgical protocols included various approaches: triple-target irradiation of the pituitary gland and both thalami (n=11, 19.6%) for mixed oncologic pain; dual-target irradiation of the trigeminal nerve and contralateral thalamus (n=35, 62.5%) primarily for chronic facial pain; bilateral thalamotomy (n=3, 5.4%); and single thalamotomy (n=9, 16.1%) for trigeminal neuralgia and other neuropathic pain syndromes (Table 1).

**Table 1:** Patient characteristics. SD, standard deviation; IQR, interquartile range; NSAIDs, non-steroidal anti-inflammatory drugs; N/A, not applicable.

Variable		Triple target (n=11)	Dual target (n=34)	Lone thalamus (n=11)		Total (n=56)
		Hypophysis + Bilateral thalamus	Trigeminal nerve + Contralateral thalamus	Bilateral thalamus (n=2)	Unilateral thalamus (n=9)	
Age (years); mean (SD)		57.9 (20.3)	57.1 (18.8)	51 (14.1)	46.3 (14.1)	55.1 (18.1)
Sex; n (%)	Male	6 (10.7%)	11 (19.6%)	0 (0%)	2 (3.6%)	19 (33.9%)
	Female	5 (8.9%)	23 (41.1%)	2 (3.6%)	7 (12.5%)	37 (66.1%)
Type of pain; n (% )	Neuropathic pain	0 (0%)	34 (60.7%)	2 (3.6%)	9 (16.1%)	45 (80.4%)
	Mixed oncologic pain (Somatic + Neuropathic/ Visceral)	11 (19.6%)	0 (0%)	0 (0%)	0 (0%)	11 (19.6%)
Duration of pain (months); median (IQR)		N/A	84 (97.5)	5.50 (10)	72 (72)	72 (96.8)
Types of medications used; n (%)	NSAIDs	5 (8.9%)	11 (19.6%)	0 (0%)	3 (5.3%)	19 (33.9%)
	Opioids	11 (19.6%)	21 (37.5%)	2 (3.6%)	8 (14.3%)	42 (75%)
	Neuromodulators	5 (8.9%)	20 (35.7%)	2 (3.6%)	5 (8.9%)	32 (66.1%)
	Antidepressants	0 (0%)	4 (7.1%)	0 (0%)	0 (0%)	4 (7.1%)
	Muscle relaxant/Local anesthetic	3 (5.3%)	1 (1.8%)	0 (0%)	0 (0%)	4 (7.1%)
Previous procedures; n (%)	None	6 (10.7%)	8 (14.3%)	0 (0%)	2 (3.6%)	16 (28.6%)
	Palliative chemotherapy	2 (3.6%)	0 (0%)	0 (0%)	0 (0%)	2 (3.6%)
	Percutaneous and/or surgical procedures	3 (5.4%)	21 (37.5%)	1 (1.8%)	3 (5.4%)	28 (50.0%)
	Stereotactic radiosurgery alone or after percutaneous or surgical procedures	0 (0%)	5 (8.9%)	1 (1.8%)	3 (5.4%)	9 (16.1%)

Oncologic pain

Demographics

Eleven patients (n=11, 19.6%) with mixed oncologic pain syndromes were treated between 2016 and 2024 using GammaRay Infini (Masep Medical Company, Shenzhen, China), Leksell Gamma Knife Icon (Elekta AB, Stockholm, Sweden), or CyberKnife (Accuray, Sunnyvale, CA, USA). The primary cancer diagnoses included adenocarcinoma of the colon (n=1, 9.1%), stomach (n=1, 9.1%), esophagus (n=1, 9.1%), pancreas (n=1, 9.1%), breast (with or without metastatic disease, n=2, 18.2%), metastatic cervical cancer (n=1, 9.1%), metastatic multiple myeloma (n=1, 9.1%), recurrent Ewing’s sarcoma (n=1, 9.1%), thigh leiomyosarcoma (n=1, 9.1%), and epidermoid carcinoma of the skin (n=1, 9.1%). All patients had completed treatment for the primary cancer, and five (n=5, 45.5%) had undergone unsuccessful procedures for pain management, including percutaneous interventions (n=3, 27.3%) and palliative chemotherapy (n=2, 18.2%). Seven patients (n=7, 63.6%) had no history of prior pain relief procedures. All were considered refractory to opioid therapy by a team of palliative care specialists and pain management experts (algologists) and were deemed unsuitable for surgical alternatives. All patients were classified as terminally ill (Table 2).

**Table 2:** Treatment characteristics. IQR, interquartile range; SRS, stereotactic radiosurgery; NRS, Numerical Rating Scale; BNI, Barrow Neurological Index; RA, right arm; RH, right hand; LL, left leg.

Variable		Triple target (n=11)	Dual target (n=34)	Lone thalamus (n=11)		Total (n=56)
		Hypophysis + Bilateral thalamus	Trigeminal nerve + Contralateral thalamus	Bilateral thalamus (n=2)	Unilateral thalamus (n=9)	
Equipment, n (%)	Gamma Knife Infini	4 (7.1%)	22 (39.3%)	2 (3.6%)	9 (15.5%)	37 (66.1%)
	Gamma Knife Icon	2 (3.6%)	1 (1.7%)	0 (0%)	0 (0%)	3 (5.4%)
	CyberKnife	5 (8.9%)	11 (19.6%)	0 (0%)	0 (0%)	16 (28.6%)
Thalamus dose (Gy); median (IQR)		90 (0)	120 (30)	125 (0)	140 (0)	120 (50)
Dose to secondary structure (Gy); median (IQR)	Trigeminal nerve	N/A	80 (21.3)	N/A	N/A	80 (25)
	Hypophysis	90 (0)	N/A	N/A	N/A	90 (0)
Pre-SRS NRS score; median (IQR)		10 (1.5)	10 (1)	10 (0)	10 (0)	10 (1)
Follow-up (d); median (IQR)		38 (44.5)	365 (356.3)	371 (289.5)	384 (413.0)	343.5 (334.5)
Post SRS NRS; median (IQR)		5 (4)	3.5 (3.8)	0 (0)	6 (9)	4 (4.3)
Time to response (d); median (IQR)		2.50 (16.3)	3 (44)	1.50 (0.5)	33 (8)	3 (32.0)
Treatment response according to reduction of initial NRS; n (%)	Complete (100% reduction)	0 (0%)	5 (8.9%)	2 (3.6%)	2 (3.6%)	9 (16.1%)
	Good (50-99%)	7 (12.5%)	21 (37.5%)	0 (0%)	2 (3.6%)	30 (53.6%)
	Poor (1-49% NRS reduction)	3 (5.4%)	3 (5.4%)	0 (0%)	2 (3.6%)	8 (14.3%)
	None (0% NRS reduction)	1 (1.8%)	4 (7.1%)	0 (0%)	3 (5.4%)	8 (14.3%)
Crisis recurrence after response (NRS 10 after 50-100% decrease); n (%)	Yes	0 (0%)	8 (14.3%)	0 (0%)	4 (7.1%)	12 (21.4%)
	No	10 (17.8%)	25 (44.6%)	2 (3.6%)	5 (8.9%)	42 (75.0%)
Time to crisis recurrence; median (IQR)		N/A	75 (315.5)	N/A	377.5 (111)	299.5 (321.8)
Severe pain recurrence after response (NRS 7-9 after 50-100% decrease); n (%)	Yes	6 (10.7%)	10 (17.9%)	0 (0%)	3 (5.4%)	19 (33.9%)
	No	4 (7.1%)	22 (39.2%)	2 (3.6%)	6 (10.7%)	34 (60.7%)
Toxicity, n (%)	Paresthesia	0 (0%)	9 (16.1%)	0 (0%)	0 (0%)	9 (16.1%)
	Headache	1 (1.8%)	0 (0%)	0 (0%)	0 (0%)	1 (1.8%)
	Alopecia	0 (0%)	1 (1.8%)	0 (0%)	0 (0%)	1 (1.8%)
	Pin perforation of the calvaria	1 (1.8%)	0 (0%)	0 (0%)	0 (0%)	1 (1.8%)
	Non-painful anesthesia	0 (0%)	1 (1.8%)	0 (0%)	0 (0%)	1 (1.8%)
	Fatigue	0 (0%)	0 (0%)	0 (0%)	0 (0%)	0 (0%)
	Sensory deficit, delayed radionecrosis and trigeminal neuritis	0 (0%)	1 (1.8%)	0 (0%)	0 (0%)	1 (1.8%)
	None	8 (14.3%)	24 (54.8%)	2 (3.6%)	9 (16.1%)	43 (76.8%)
Radiomodulation effect, n (%)	Yes	7 (12.5%)	23 (41.1%)	2 (3.6%)	1 (1.8%)	33 (58.9%)
	No	4 (7.1%)	11 (17.9%)	0 (0%)	8 (14.3%)	23 (41.1%)

Pre-radiosurgery NRS scores averaged 10 for all oncologic pain patients. Medication regimens included opioids (n=11, 100%), neuromodulators (n=5, 45.5%), nonsteroidal anti-inflammatory drugs (NSAIDs, n=5, 45.5%), local anesthetics (n=3, 27.3%).

Follow-Up

Follow-up assessments were conducted via telemedicine, using phone calls, video calls, or voice messaging, and included evaluations of pain intensity, daily frequency of pain episodes, episode severity, pain triggers, medication use, and the impact of pain on daily activities. The NRS and the EQ-5D scale were used to assess health-related quality of life. When possible, data were collected directly from the patients; otherwise, primary caregivers provided the information. Follow-up occurred daily during the first week and then weekly until the patient’s death.

Radiosurgical Planning

Treatment was delivered using GammaRay Infini (Masep Medical Company, Shenzhen, China) for four patients (n=4, 36.4%), CyberKnife (Accuray, Sunnyvale, CA, USA) for five patients (n=5, 45.5%), and Leksell Gamma Knife Icon (Elekta AB, Stockholm, Sweden) for two patients (n=2, 18.2%). For mixed oncologic pain, the targets were the pituitary gland and both thalami for all patients. A dose of 90 Gy was administered to all structures.

Pain Outcomes

Substantial, quick pain relief was observed in seven patients (n=7, 63.6%) with oncologic pain. The median time to response was 2.5 days. A desirable treatment response was defined as a reduction of at least 50% in the original NRS score compared to pre-radiosurgery levels. Responses were further classified into "complete" for a 100% reduction of NRS scores, "good" for a reduction of 50% to 99% in NRS scores, "poor" with a reduction of 1 to 49%, and no response as 0% reduction.

**Figure 1:**
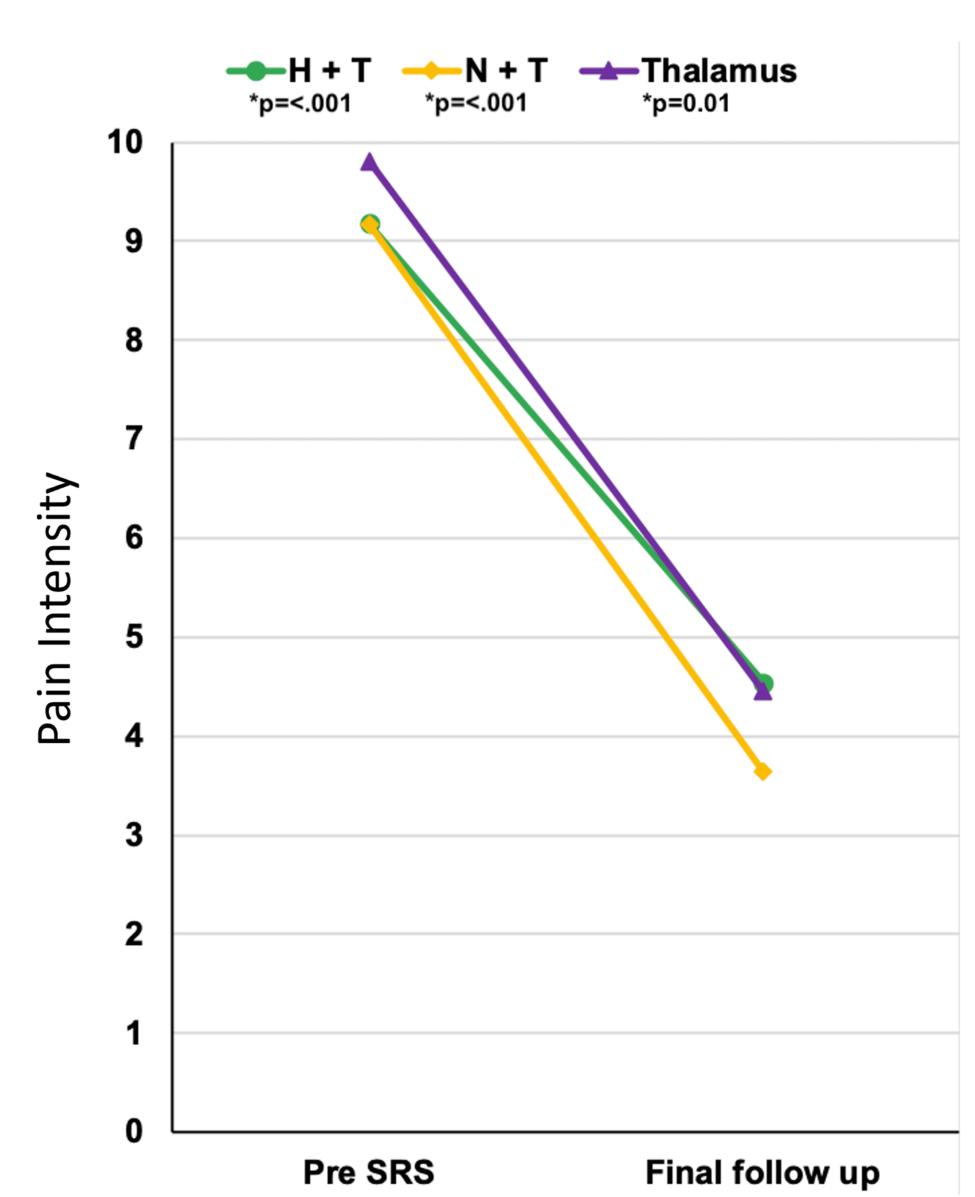
Mean pain intensity according to NRS scores before and after treatment Comparison of mean pain intensity (NRS) before and after treatment for all intervention groups. Pain relief was statistically significant for triple-target radiosurgery (Student’s t-test, p < 0.001), dual-target radiosurgery (Wilcoxon test, p < 0.001), and single/bilateral thalamotomy (Wilcoxon test, p = 0.01). NRS, Numerical Rating Scale; SRS, stereotactic radiosurgery.

**Figure 2:**
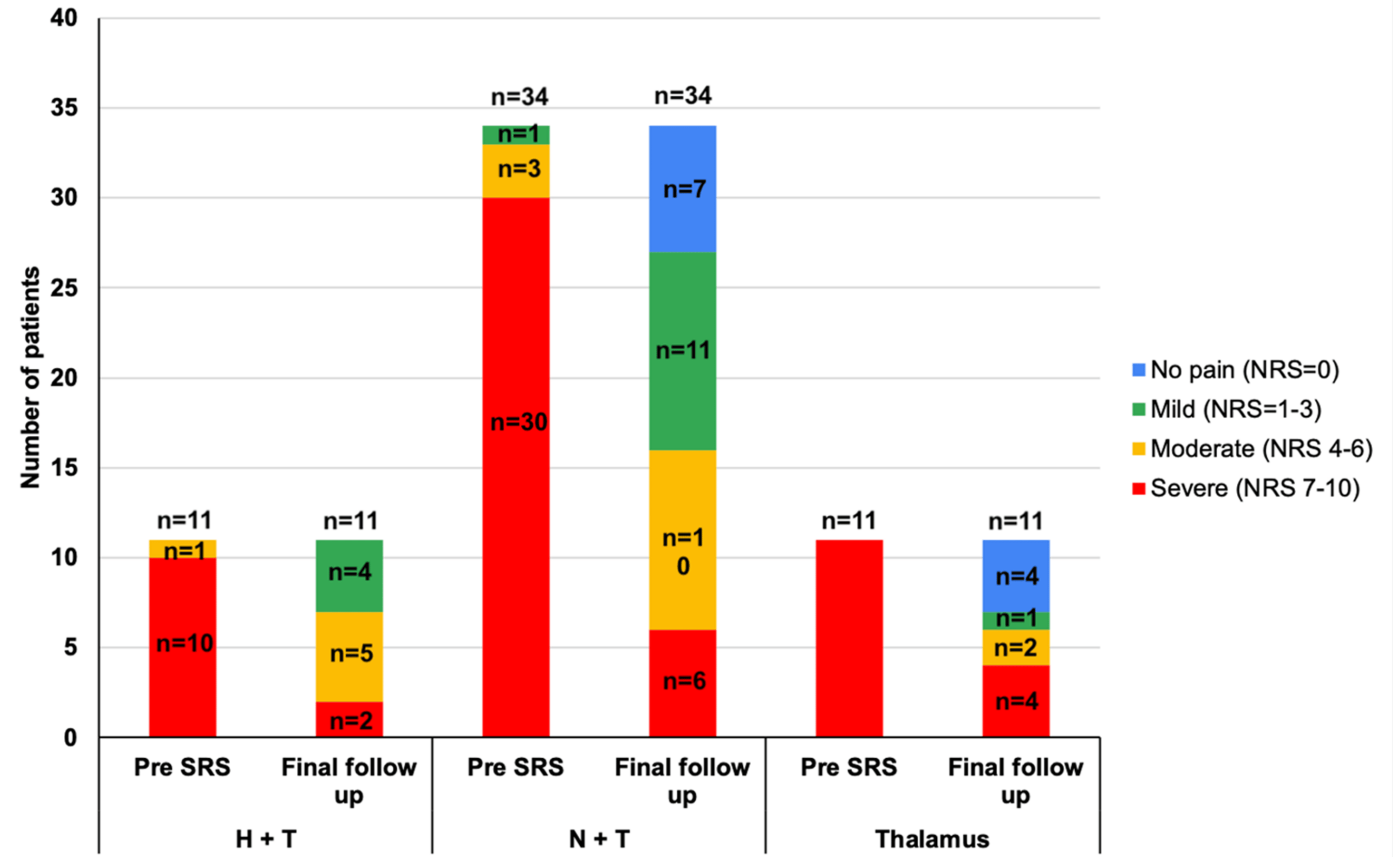
Evolution of pain severity according to NRS scores across treatment groups Pain severity was stratified according to NRS scores before and after SRS, with NRS=0 equal to no pain, NRS=1-3 classified as mild pain, NRS=4-6 as moderate pain, and NRS=7-10 as severe pain. NRS, Numerical Rating Scale; SRS, stereotactic radiosurgery.

Complete resolution of pain (NRS score of 0) was not achieved in the oncologic pain group. However, seven patients (n=7, 63.6%) experienced a “good” response, defined as a reduction of 50% to 99% in NRS scores; three patients (n=3, 27.3%) showed a “poor” response, with a reduction of 1% to 49%; and one patient (n=1, 8.3%) had no response. Recurrence of severe pain crises (NRS=10) did not occur; however, six patients (n=6, 54.5%) reported severe pain (NRS 7 to 9) after initial improvement. Before treatment, the median NRS score was 9 (range, 6 to 10), which decreased to 4.5 (range, 2 to 8) at the last follow-up (Student’s t-test, p < 0.001). Among the 10 patients (n=10, 90.9%) who had died by the time of the last follow-up, the mean survival duration was 66.8 days (Figures 1, 2).

Toxicity

Adverse effects in patients with oncologic pain were minimal. One patient (n=1, 9.1%) reported headache, while another (n=1, 9.1%) experienced both headache and fatigue. One patient sustained a pin perforation injury to the skull during head frame placement due to weakened bone structure from the primary cancer. The patient was treated with oral antibiotics and monitored closely, without developing any major complications.

Neuropathic pain

Demographics

Between 2016 and 2024, 45 patients with neuropathic pain were treated with radiosurgery. The GammaRay Infini (Masep Medical Company, Shenzhen, China) was used in 34 patients (n=34, 73.3%), Leksell Gamma Knife Icon (Elekta AB, Stockholm, Sweden) in one patient (n=1, 2.2%), and CyberKnife (Accuray, Sunnyvale, CA, USA) in 12 patients (n=12, 26.6%). Follow-up was available for all 45 patients.

The average duration of pain was 90.4 months. Of all neuropathic pain patients, 25 patients (n=25, 55.5%) had previously undergone percutaneous and/or surgical procedures for pain relief. Ten patients (n=10, 20%) had SRS after unsuccessful percutaneous or surgical interventions, defined as no pain relief within six months post-procedure. Ten patients (n=10, 22.2%) had no history of prior procedures. The most common diagnosis was refractory type I trigeminal neuralgia (n=19, 42.2%), followed by atypical trigeminal neuralgia (n=12, 26.6%) and anesthesia dolorosa secondary to trigeminal neuralgia treatments (n=4, 8.8%). Other conditions included post-herpetic or post-traumatic trigeminal neuralgia (n=3, 6.6%), trigeminal neuralgia secondary to tumor (n=2, 4.4%) or basilar dolichoectasia (n=1, 2.2%), atypical facial pain (n=1, 2.2%), deafferentation pain (n=1, 2.2%), idiopathic trigeminal neuralgia (n=1, 2.2%), and complex regional pain syndrome (CRPS) (n=1, 2.2%).

Pharmacological management included opioids in 68.8% (n=31) of patients, neuromodulators in 60.0% (n=27), NSAIDs in 31.1% (n=14), and other medications such as antidepressants or muscle relaxants in 2.2% (n=1). The mean pre-SRS NRS score was 9.3 for all neuropathic pain patients, 9.1 for those treated with dual-target radiosurgery, 9 for patients with bilateral thalamotomy, and 10 for single thalamotomy.

Follow-Up and Pain Assessment

The NRS, EQ-5D for health-related quality of life, and BNI for trigeminal pain were used for assessments. For patients with chronic continuous pain (CCP) and paroxysmal pain, an average NRS score was calculated for both crisis and baseline CCP pain. The total hours spent in pain and crisis per day were used to provide an overall average NRS score, which was reported separately.

Follow-up assessments were conducted daily during the first week, at two weeks, monthly for the first year, and annually thereafter when possible. Patients were instructed to continue their pain medications as their primary care physicians directed.

Radiosurgical Planning

Treatment was delivered using the GammaRay Infini (Masep Medical Company, Shenzhen, China) for 33 patients (n=33, 73.3%), CyberKnife (Accuray, Sunnyvale, CA, USA) for 11 patients (n=11, 24.4%), and Leksell Gamma Knife Icon (Elekta AB, Stockholm, Sweden) for one patient (n=1, 2.2%). Targets for neuropathic pain included dual-target irradiation of the trigeminal nerve and contralateral thalamus (n=34, 75.5%), bilateral thalamotomy (n=2, 4.4%), and single thalamotomy (n=9, 20.0%). For the triple target group, a 4 mm collimator was used with Infini and a 5 mm cone with CK, and a single shot with a Dmax of 90 Gy was placed bilaterally in each region of the thalamus, considering the 20 Gy isodose line as a possible “area of influence” of radiation. For the hypophysis, the neurohypophysis was defined as the isocenter of the shoot, using an 8 mm collimator with Infini and a 7.5 mm collimator with CK to deliver a dose of 90 Gy Dmax; the complete optic pathway and brainstem were defined as an organ at risk so that in any case the dose would not be superior to 8 Gy in the optic pathway or 15 Gy at a focal point in the brainstem. For the CK, an isocentric 90 Gy to 97% plan was created, using a short path and Monte Carlo algorithm at high resolution for dose calculations, and 6D skull tracking was used as standard. For the dual target group, a 4-mm collimator was used with Gamma Knife (GK) and 5 mm for CyberKnife (CK), placing a single shot administering 80 to 140 Gy as a maximum dose (Dmax). Additionally, patients in this group were prescribed an 80 to 90 Gy Dmax using a single 4 mm (GK) or 5 mm (CK) isocenter positioned in the retrogasserian zone of the affected nerve.

**Figure 3:**
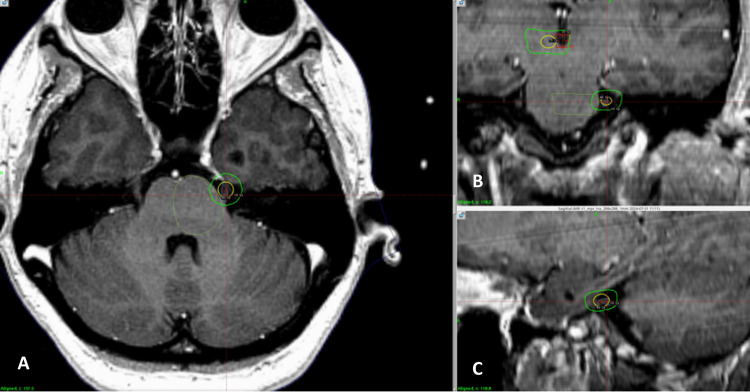
Three-dimensional view of a Gamma Knife treatment for refractory chronic facial pain. Treatment plan in Gamma Knife for refractory neuropathic facial pain. The left trigeminal nerve in the retrogasserian portion is targeted using a 4-mm collimator with a prescribed dose of 80 Gy. The yellow isodose line represents 50% of the prescribed dose (40 Gy), and the outermost isodose line shows 10 Gy. In the superior right coronal image, the 50% isodose line (70 Gy) is seen in yellow in the right thalamus, and the outermost green isodose line indicates 10 Gy.

For dual-target treatments, median doses were 80 Gy to the trigeminal nerve and 120 Gy to the contralateral thalamus. Median thalamic doses were 140 Gy for single thalamotomy and 125 Gy for bilateral thalamotomy (Figure 3).

Pain Outcomes

Substantial, quick pain relief was observed in 26 patients (n=26, 57.7%) with neuropathic pain, of which 23 (n=23, 51.1%) belonged to the dual-target group, two (n=2, 4.4%) to the bilateral thalamotomy group, and one (n=1, 2.2%) to the single thalamotomy group. A treatment response was defined as a reduction of at least 50% in the original NRS score. Median time to response was three days for dual-target SRS, 1.5 days for bilateral thalamotomy, and 33 days for single thalamotomy.

**Figure 4:**
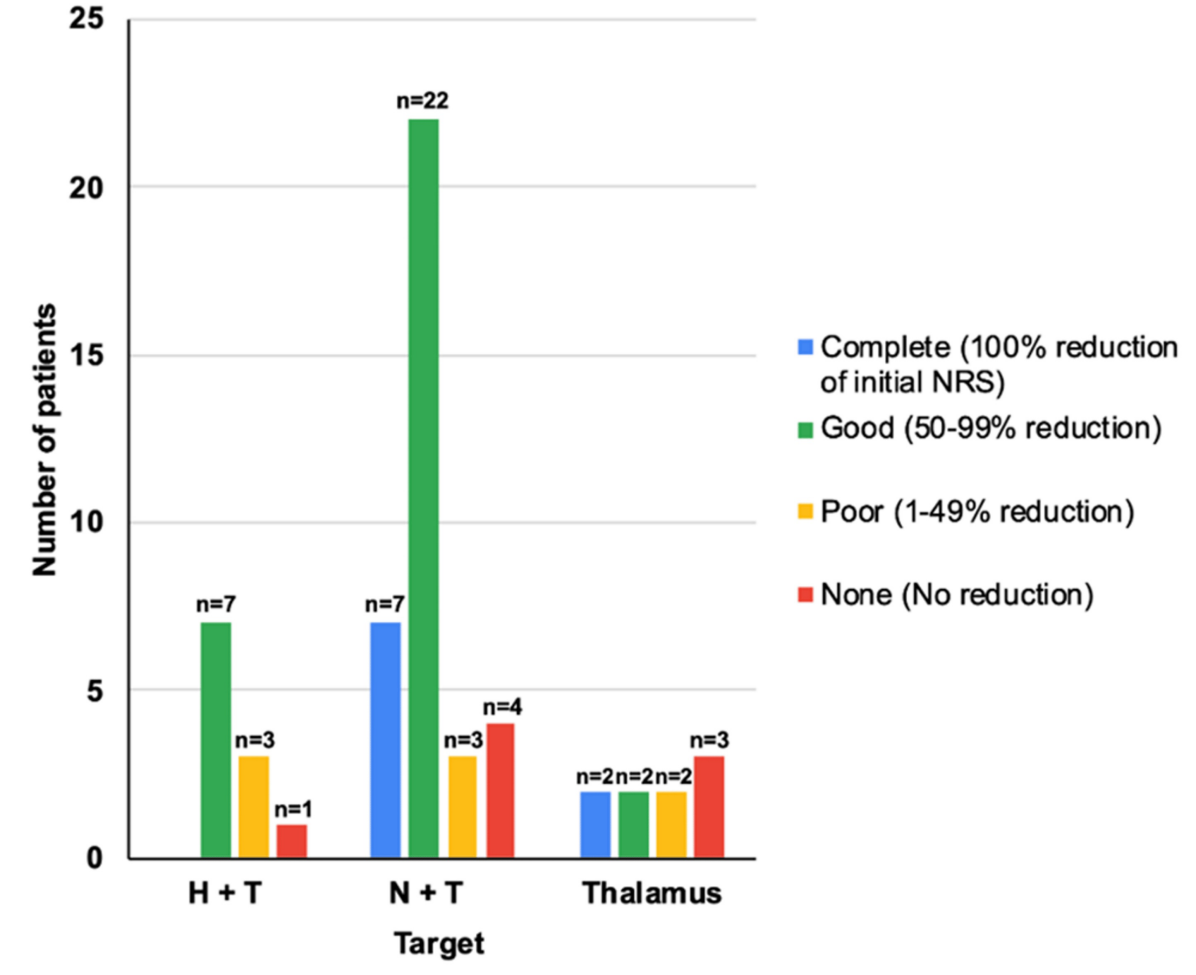
Final treatment response shown as the percentage reduction in initial NRS scores Treatment responses were classified according to the percentage of reduction in final NRS scores as compared to initial scores. NRS, Numerical Rating Scale.

Treatment responses were classified correspondingly to the oncological pain group. At the final follow-up, nine patients (n=9, 20%) achieved complete pain relief (NRS score of 0), including five who underwent dual-target irradiation, two with single thalamotomy, and two patients treated with bilateral thalamotomy. Of the 23 patients (n=23, 51.1%) who experienced a “good” response (50% to 99% reduction in NRS scores), 21 (n=21, 46.6%) were in the dual-target group, and two (n=2, 22.2%) were in the single thalamotomy group. A “poor” response, defined as a 1% to 49% reduction in NRS scores, occurred in five patients (n=5, 11.1%): three from the dual-target group and two from the single thalamotomy group. Seven patients (n=7, 15.5%), including four with dual-target treatment and three with single thalamotomy, experienced no pain relief, resulting in a 26% failure rate for pain relief (Figure 4).

Following initial response, eight patients (n=8, 17.7%) in the dual-target group and four patients (n=4, 44.4%) in the unilateral thalamotomy group experienced severe pain crises (NRS=10) before achieving sustained relief at six months post-SRS. Patients treated with bilateral thalamotomy did not report any pain exacerbations post-treatment. Recurrence of severe pain (NRS 7 to 9) was noted in 10 dual-target patients (n=10, 22.2%) and three single thalamotomy patients (n=3, 33.3%). No recurrences were reported in the bilateral thalamotomy group at the last follow-up. Overall, pain recurrence for the series was 31.3%. Median NRS scores decreased from 9 (range 4-10) before treatment to 3.8 (range 0-10) at the last follow-up for dual-target and single/bilateral thalamotomy patients (Wilcoxon test, p < 0.001 and p = 0.01, respectively). The mean follow-up duration was 410.4 days (range 30-994 days). Overall treatment success, defined as at least 50% pain relief, was 69.1% for the entire cohort, with a success rate of 66.6% for oncologic pain and 71.7% for non-oncologic pain.

**Figure 5:**
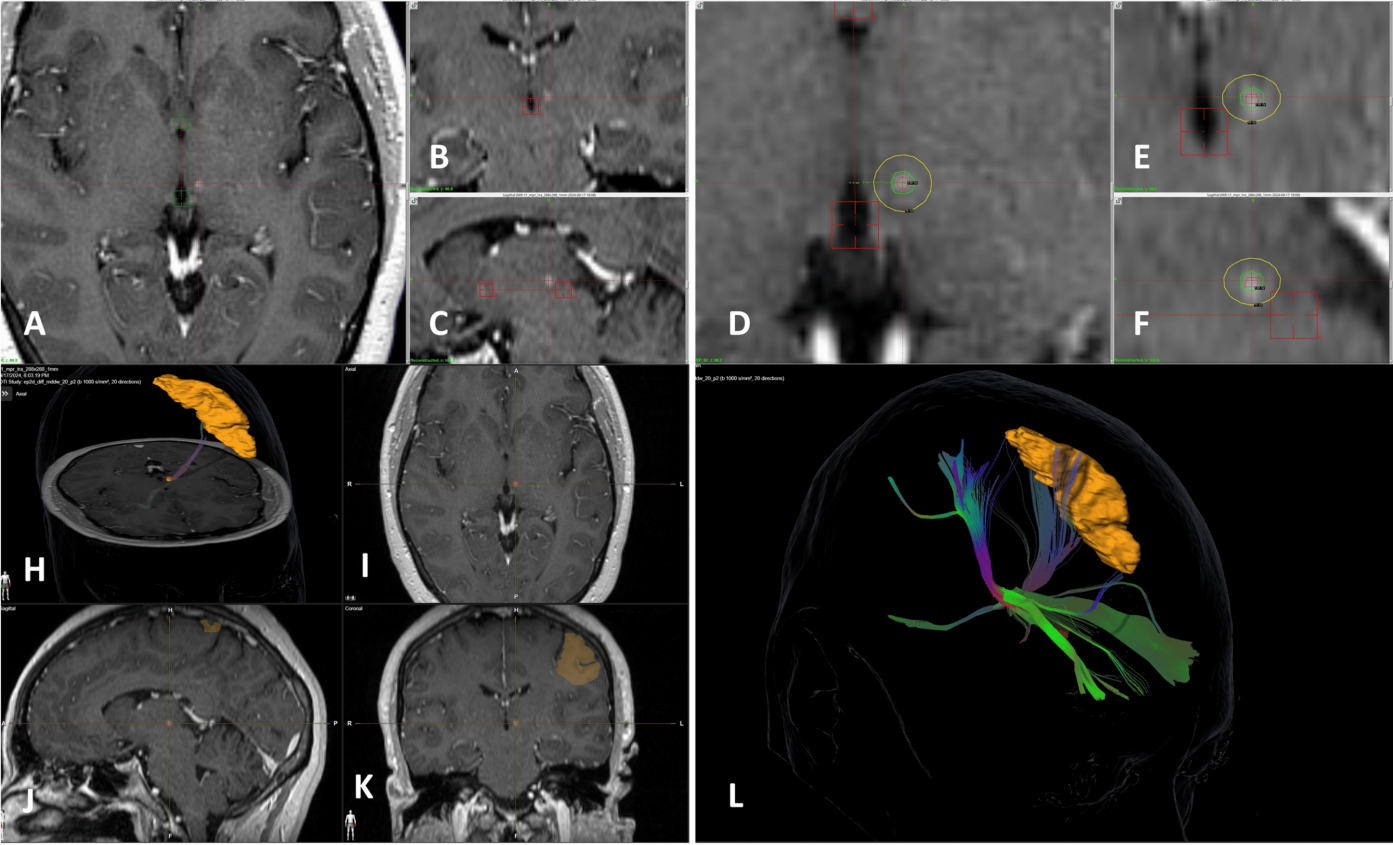
Three-dimensional view of a left thalamotomy (140 Gy) 18 months post-radiosurgery, showing contrast enhancement in the central structures of the left thalamus. Close-up three-dimensional view with an overlay of a 4 mm 140 Gy Gamma Knife shot targeting the enhancing area in the left thalamus. The internal green isodose line encircling most of the enhancement represents the 130 Gy isodose, while the outer yellow isodose line indicates 70 Gy. Three-dimensional representation of spinothalamic fibers within an ROI limited to the contrast-enhancing area. The orange region at the cortex represents the postcentral gyrus, also known as the primary sensory cortex. Three-dimensional reconstruction showing the fibers in both thalami, with an ROI extending to the 70 Gy isodose line. The orange area at the cortex represents the left postcentral gyrus. Brainlab Elements (Brainlab, Munich, Germany) was used to generate images in Figure 5H and Figure 5L. ROI, region of interest.

Contrast enhancement was evidenced in the central structures of the left thalamus by a neuropathic pain patient 18 months post-radiosurgery. Three-dimensional representation of spinothalamic fibers within an ROI limited to the contrast-enhancing area, which affected the postcentral gyrus, also known as the primary sensory cortex, within the 130 Gy isodose line. Three-dimensional reconstruction of the fibers in both thalami showed an ROI extending to the 70 Gy isodose line, extending to the left postcentral gyrus (Figure 5).

Toxicity

**Figure 6:**
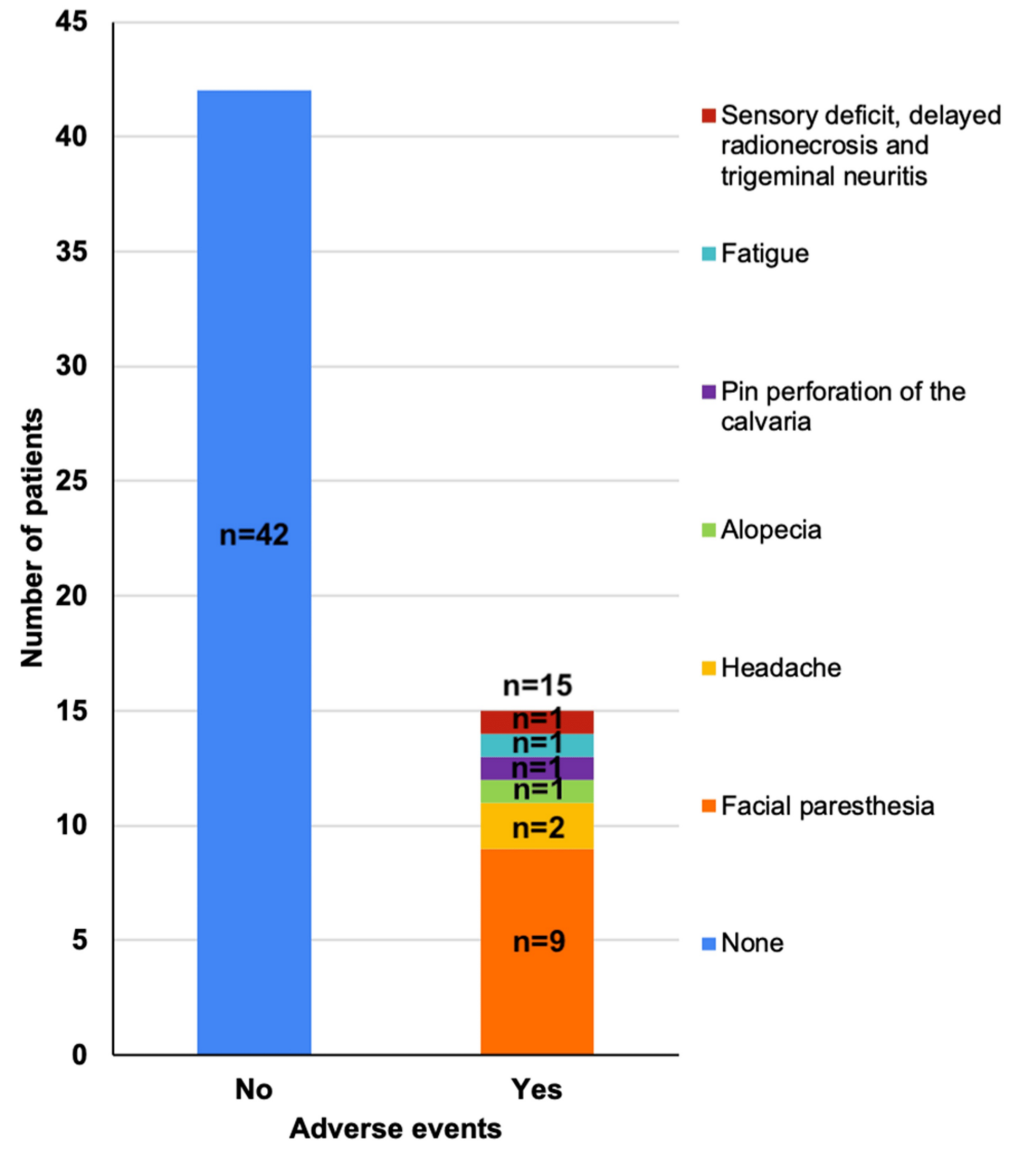
Adverse events associated with radiation treatment SRS, stereotactic radiosurgery

Most patients experienced mild, transient side effects. Nine patients (n=9, 20%) reported non-bothersome facial paresthesia, all of whom were in the dual-target group. One patient experienced both facial paresthesia and alopecia (n=1, 2.2%). One patient in the dual-target group developed a sensory deficit, delayed radionecrosis, and trigeminal neuritis eight months after the procedure, which was managed appropriately. This patient was treated using the CyberKnife system (Figure 6).

## Discussion

Pain involves three pathways: the lateral, medial, and descending pathways. The lateral pathway processes the type, intensity, and location of pain, while the medial pathway, which projects to the limbic system, mediates the emotional aspects of pain, including suffering. The descending pathway is responsible for regulating pain. Maladaptive changes in these pathways can lead to chronic pain, a complex condition that significantly impairs quality of life. The chronification of pain is associated with changes in brain networks at an executive level, reflecting the transformation from acute to chronic pain. Understanding these physical and biochemical changes is crucial for effectively managing chronic pain through interventions targeting different levels of the pain pathways [8].

For a large proportion of patients, even optimal medical treatments may not provide sufficient relief, and surgical options may not be viable, especially in terminally ill cancer patients [6]. In cases of chronic facial pain, trigeminal neuralgia can result from trigeminal nerve injury, whether due to surgical procedures or nerve encasement by tumors. These conditions can limit the feasibility of traditional approaches such as microsurgical decompression, radiosurgery, or percutaneous rhizotomy [9]. When the trigeminal nerve is not a viable target, SRS to the thalamus may offer an alternative treatment for these challenging cases.

The medial thalamus is well established as a target for refractory pain, both neuropathic and oncologic, due to its central role in pain perception and the emotional response to pain. It has been a focal point for deep brain stimulation, radiofrequency ablation, radiosurgery, and high-frequency ultrasound lesioning [2,3]. However, the clinical outcomes of radiosurgical thalamotomy are highly variable, with pain relief effects often diminishing over time [7, 10, 11]. This variability may be explained by the bilateral nature of pain representation and the complex network changes within the limbic system [8].

Within the medial thalamus, the sensorimotor nuclei, including the centromedian, parafascicular, and central lateral portions of the mediodorsal nucleus, are considered part of the limbic system and are directly involved in the emotional dimensions of pain [12,13]. In this study, the irradiated areas, based on the cockade model [7, 14], included regions within the 20 Gy isodose line. This area encompasses the parvocellular portion of the ventroposteromedial (VPM) nucleus dorsally and the centromedian-parafascicular (CM-Pf) complex ventrally. According to our group's recent structural and connectivity analyses, these regions also involve portions of the periaqueductal gray, a key component of the descending pain modulation pathway (unpublished data). Thus, 20 Gy or higher radiation doses can cover all three pain pathways.

The VPM serves as a relay center for the trigeminal principal sensory nucleus and the spinal nucleus, which receive direct afferent signals from the spinal trigeminal tract and project to the spinothalamic pathway. Neurons in this nucleus transmit these signals somatotopically to the postcentral gyrus in the primary sensory cortex. The VPM also conveys slow, dull pain signals to the contralateral reticular formation, relaying the information to the intralaminar thalamic nuclei, including the CM-Pf complex, and throughout the cerebral cortex. This widespread transmission contributes to the diffuse nature of chronic pain, making it challenging to localize [8, 15]. As shown in Figure 5, the fibers at the thalamic target, as depicted in reconstructed diffusion tensor imaging, correspond to the area targeted for irradiation in our study; projections can be seen to the main sensory and prefrontal cortex.

The CM-Pf complex, part of the medial thalamic nuclei, receives input from the spinothalamic tract and trigeminal lemniscus and projects to the cingulate cortex. The centromedian nucleus is extensively connected to the limbic-associative system, playing a significant role in pain perception and its affective components. The parafascicular nucleus is also potentially linked to the periaqueductal gray, which is involved in pain modulation [13].

Thalamotomy for oncologic pain

Stereotactic medial thalamotomy has been employed for managing malignant pain since the early days of radiosurgery, achieving significant initial relief in approximately 35% to 67% of patients [3]. However, the duration of this effect is highly variable; some patients maintain pain relief until death, while pain recurs in about 58% to 91% of those who initially respond, particularly in those with longer survival times [3,5]. To achieve consistent pain relief, other radiosurgical targets have been explored, with pituitary irradiation generally considered the most effective approach for alleviating cancer-related pain. Radiosurgical irradiation of the pituitary gland is thought to provide pain relief through a mechanism different from thalamic targets, likely involving a radio-endocrine-modulatory effect [16, 17]. This may involve the redirection of hormones such as oxytocin or proopiomelanocortin (a precursor of β-endorphin) synthesized by neurons in the adenohypophysis and hypothalamus [2], which can suppress severe pain similarly to morphine [18, 19].

Unlike invasive methods for pituitary lesioning, radiosurgical irradiation with modern doses (140-150 Gy) appears to produce minimal clinical signs of endocrine deficiency, such as hypopituitarism. Thus, the pain relief is thought to result from a stimulating effect [18]. Although reports on this procedure have been sporadic over the years, recent series indicate that endocrine disturbances are not a significant side effect in terminally ill cancer patients [16].

Despite these advantages, our experience with pituitary irradiation for malignant pain has shown recurrence rates as high as 50% by the time of death. Although this outcome is less than ideal when the goal is palliative, subsequent pain crises were generally more manageable. Our preliminary data suggest that mixed oncologic pain may not respond as well as somatic pain, as radiosurgery to the pituitary gland - and surgical hypophysectomy - was initially developed for pain related to bone metastases [3,16-19].

To address the limitations of each intervention, we propose a multi-target approach tailored to the type of pain [17,19]. For malignant somatic pain, we recommend pituitary irradiation alone. For mixed oncologic pain, we suggest combining pituitary and bilateral thalamic irradiation, using lower doses (90 Gy) for each structure to leverage the pituitary irradiation's radio-endocrine-modulatory effect and thalamic SRS's durability. This approach possibly addresses the neuropathic and visceral components of pain [17].

Our findings indicate that a triple-target irradiation strategy can produce rapid pain relief - often within 24 hours - in terminally ill patients with mixed oncologic pain, sustaining lower NRS scores until death. Additionally, this approach was associated with a significant reduction in medication use. Patients unable to reduce medication usage reported improved responses to their current treatments. Currently, a clinical trial comparing this triple-target strategy with a sham treatment for mixed oncologic pain is ongoing. While triple-target radiosurgery is a novel approach, more studies are needed to establish its long-term safety and efficacy. In this study, side effects were rare, with only one patient reporting mild and transient headache and fatigue. Patients who do not respond within one to two weeks are unlikely to improve later, suggesting that a pain response within 72 hours is predictive of treatment success.

Patient selection and management

Appropriate patient selection is crucial for the successful management of refractory pain. Oncologic pain patients should be evaluated by a multidisciplinary team, including specialists in oncology, pain management (algology), palliative care, and neurosurgery. Ideally, patients should have completed all available treatments for their primary cancer and be confirmed as refractory to other pain management options. Continuous evaluation by palliative and pain management teams is essential, and long-term follow-up is necessary to manage recurrent pain crises or potential side effects.

Thalamotomy for neuropathic pain

Thalamotomy is considered more effective for neuropathic pain, particularly upper body pain, and may yield better outcomes in patients with chronic facial pain [7, 11, 20, 21]. Our initial experience with single thalamotomy showed mixed results, with 50% of patients achieving sustained pain relief at the final follow-up. This initial series, included in the current study, consisted of patients with trigeminal neuralgia who did not respond to nerve irradiation after six months, had intracranial nerve sectioning, or had no visible nerve due to tumors. The series also included patients with anesthesia dolorosa, bilateral occipital neuralgia, post-stroke thalamic pain, and CRPS.

From this experience, we found that unilateral thalamotomy of the medial thalamic nuclei is most effective for facial pain and may not be a viable option for C2 neuralgia, localized pain, or other neuropathic syndromes when used unilaterally. Franzini et al. reported using bilateral thalamotomy with Gamma Knife radiosurgery, targeting a more lateral area, for such cases [21].

Early responses to radiation

Early responses to radiation in the unilateral thalamotomy group were not well documented initially, as we did not fully understand the clinical effects observed and considered them potentially due to a placebo effect. According to existing literature, therapeutic responses to radiation were believed to be associated with the slow formation of a necrotic lesion, with a latency of at least three months [22]. Significant pain relief was therefore not expected during this latency periods, however, reports of rapid, sustained, and significant pain relief achieved with focal radiation suggested a neuromodulatory effect of radiation (RM) [20]. This clinical effect has been defined by some authors as a neuronal circuit alteration induced by sub-ablative doses of radiation [22, 23].

To explain this phenomenon, Régis proposed a possible radiomodulatory effect for epilepsy relief observed in patients treated for arteriovenous malformations, even before significant radiological changes in the vascular anatomy were apparent [14]. The group introduced a cockade model to describe this effect, suggesting that focally delivered radiation creates three concentric zones. The innermost zone is necrotic, surrounded by a sub-necrotic area characterized by cellular death without coagulative necrosis, and an outer neuromodulatory zone where subtle inflammatory changes occur without increased cellular death. The effects in each zone can be influenced by adjacent areas, extending the impact beyond the central lesion.

We aimed to induce substantial, quick relief in refractory patients experiencing acute neuropathic pain crises, where rapid pain relief is especially desirable. Early responses in such patients have been reported following irradiation of the medial thalamus and the trigeminal nerve [20, 23, 24]. Despite notable initial pain relief, this analgesic effect can be temporary, with permanent relief developing over weeks to months. The timing of the final response is variable and may coincide with the appearance of a contrast-enhancing lesion, which is empirically linked to the necrotic effect. In our study, this variability was evident: unilateral thalamotomy had a median time to relief of 36 days, while bilateral thalamotomy showed a quicker median time of 1.5 days, suggesting that intervening at multiple targets may accelerate pain relief compared to a single target.

In a subsequent publication, early relief was more reliably achieved with higher radiation doses to the thalamus and extended treatment times, independent of the biologically effective dose (BED) [23]. Longer treatment durations with the same physical dose resulted in a lower BED compared to treatments delivered in shorter time frames, a phenomenon better understood with Gamma Knife technology due to cobalt decay. BED should be considered when comparing outcomes across different technologies. In our research, longer exposure to radiation and higher physical doses were associated with more significant results, supporting the hypothesis that prolonged radiation exposure may influence neuronal circuit outcomes. The practical explanation for why higher doses provide faster pain relief is that larger thalamic areas receive high doses (>20 Gy), potentially inducing a neuromodulatory effect.

In patients with trigeminal neuralgia, nerve irradiation can induce substantial, quick relief by decreasing ephaptic transmission, promoting axonal degeneration, and altering surviving neurons, even at sub-ablative doses (40 to 60 Gy) [22, 23]. However, using radiosurgery to target the nerve, we achieved this effect in only 16% of patients. Early pain relief correlated with the targeted zone: the retrogasserian zone showed significant relief after a mean of 22.3 days, while the dorsal root entry zone took a mean of 34.1 days [25].

Dual-target radiosurgery: nerve and thalamus

Concomitant irradiation of the trigeminal nerve and thalamus was first proposed by Keep et al. in a small case series involving three patients with post-herpetic trigeminal neuralgia, aiming to address both abnormal peripheral input and its central amplification, with a reported 60% success rate [15,26]. Our experience with this dual-target approach indicates that radiation doses of at least 120 Gy, ideally 140 Gy, to the medial thalamic nuclei combined with nerve irradiation provide significantly improved pain control compared to lower doses. Pain control rates more than doubled at three months, and a 70% control rate was achieved at one year for chronic facial pain (unpublished data) (Table 3).

**Table 3:** Published articles describing radiosurgical thalamotomies for pain. TN, trigeminal neuralgia; SRS, stereotactic radiosurgery; VAS, Visual Analogue Scale; BNI, Barrow Neurological Index; RNM, radio-neuromodulation effect; IQR, interquartile range; GKRS, Gamma Knife Radiosurgery; RZN, retrogasserian zone; MRI, magnetic resonance imaging.

Authors	Year	Article type	Inclusion criteria	Platform	Patients (n)	Dose	Target	Follow up (m)	Results
Lovo et al. [7]	2019	Retrospective	Refractory trigeminal neuralgia, anesthesia dolorosa secondary to surgical procedures, nerve sectioning.	Infini™ stereotactic frame (Masep Medical Company, Shenzhen, China)	10	140 Gy	Centromedian parafascicular complex (CM-Pf) of the contralateral thalamus	12 (1-32)	Radio-neuromodulation (Reduction ≤25% of pain in 1-30 days) in 60% patients (8.5 d, 2-15) (VAS=3,: 0-7). Total success (BNI I-IIIb) of 60%. BNI I in 30% (n=3), BNI IIIa-IIIb in 30%. One case within the success group recurred after 798d. Reduction of ≥25% monthly visits to the emergency room in 90%, with a reduction of at least one medication, even in those with treatment failure (BNI IV-V).
Urgosik and Liscak [10]	2018	Retrospective	Refractory thalamic pain, postherpetic TN, causalgic, phantom pain trigeminal deafferentation pain or neuralgia.	Leksell Gamma Knife	30	145-150 Gy	Centromedian and parafascicular complex (CM-Pf) of the contralateral thalamus	12-18	Overall success of 43.3% of patients and failed in 56.7% of patients. The duration of pain reduction was 10–72 months; in 4 patients pain relief was interrupted by recurrence. Pain recurred in 4 (31%) of 13 patients between 22 and 30 months after irradiation. There were no reports of new neurological impairment, worsening of existing neurological deficits, or worsening of pain symptoms.
Keep et al. [15]	2005	Prospective	Patients with severe refractory postherpetic trigeminal neuralgia	Model B Leksell Gamma Knife unit (Elekta Instrument AB).	3	120-140 Gy (T); 60-80 Gy (N)	Centromedian nucleus of thalamus, retrogasserian zone of trigeminal nerve.	6 to 53	Case 1 had no severe attacks of pain (7/10) and reduced dose of nortriptyline by half within 1 month, with relief of 75% for 53m. Case 2 had decreased intensity (2/10) without medication at 1 w. Relief of facial pain >50% persisted until his cancer-related death at 6m. Case 3 reported reduction in the size of the affected area at month 6.
Lovo et al. [17]	2022	Retrospective	Terminally ill patients with complex cancer pain syndromes, refractory to opioids and invasive treatment	Infini™ stereotactic frame (Masep Medical Company, Shenzhen, China), Cyberknife (Accuray, Sunnyvale, CA, USA)	3	90 Gy to each structure	Bilateral centromedian parafascicular complex (CM-Pf) + Neurohypophysis	1 (until death)	Significant VAS reduction (VAS 4, 5, and 6 compared to previous 10) within 96 hours. Improvement of response to medical management and significant pain reduction until time of death (VAS 0, 2, 6) with significant reduction of morphine rescues in all patients (70-84%). Mild fatigue reported after SRS.
Lovo et al. [20]	2022	Retrospective	Refractory TN with a severe crisis (VAS= 10) ≥ 4 weeks, refractory to opioids and invasive treatment, without other treatment options	Infini™ stereotactic frame (Masep Medical Company, Shenzhen, China)	8	80-90 Gy (Nerve) + 120-140 Gy (Thalamus)	Retrogasserian zone (RZN) of the affected nerve + contralateral centromedian parafascicular complex (CM-Pf)	2-7	RNM effect (VAS score reduction of ≥50% within 72 hours) in all patients in 48 h, VAS score: 2 (0-3). At 3 months, 12.5% (n=1) had BNI I, and 87.5% (n=7) had BNI IIIa, IIIb. Total success rate was 100% (BNI I to IIIb). 25% (n=2) had moderate pain at last follow-up (VAS 4-7), but had become responsive to medication. 75% (n=6) reported no pain to mild pain at last follow-up. No complications, including facial paresthesia, were documented. 37.5% (n=3) reported pain crises (VAS 8-10) within the follow-up period, managed with medications.
Franzini et al. [21]	2020	Prospective	Chronic, refractory neuropathic pain refractory treated with GKRS ablation of the thalamic CLp	Leksell Gamma Knife Perfexion™ (Elekta Instruments AB)	8	130-140 Gy	Central lateral nucleus of the thalamus (CLp), either unilaterally or bilaterally	12-36	By 24-36 months, 5/8 patients reported 50% VAS score reduction, mean=9.5-4.5. After 1 year the mean VAS pain score decreased significantly, from 9.4 (range 8–10) to 5.5 (mean -41.33%, p = 0.01). Statistically significant improvement of the SF- 36v2 quality of life survey (mean +48.16%, p = 0.012) and EQ-5D (+45.16%, p = 0.012) was observed. By 2 years, VAS scores were significantly reduced at 5.5 in 6/8 patients. Pain recurred in two patients, diagnosed with central post stroke neuropathic pain and brachial plexus injury.
Moreira et al. [23]	2024	Retrospective	Refractory trigeminal neuralgia with severe, continuous concomitant pain that persisted ≥ 4w.	Gamma Ray Infini (Masep Medical Company, Shenzhen, China), Cyberknife (Accuray, Sunnyvale, CA, USA)	21	High >120 Gy T + 90 Gy N. Low <120 Gy T, >90 Gy N	RZN of the trigeminal nerve + contralateral CM-Pf	3	High dose group showed radiomodulation effect at 1d, low dose group did not produce radiomodulation on 4d. VAS score reduction was higher in the high thalamus dose group than in the low-thalamus dose group at 1m. VAS score was 2 in the high-dose group and 4 in the low dose group at 3m.
Franzini et al. [24]	2022	Retrospective	Intractable chronic neuropathic pain syndromes treated with stereotactic CL thalamotomy	Leksell Gamma Knife models Perfexion™ and Icon™ (Elekta)	21	130-140 Gy	Central lateral nucleus of the thalamus, either unilaterally or bilaterally	12-48	Meaningful initial pain reduction, (VAS decrease ≥50% and BNI I-III status), was achieved in 12 patients (57%) after a median of 3 months (IQR 3-6 months), more frequently in patients with trigeminal deafferentation pain (83% vs 22%, P = .009). Pain recurred in 5 initial responders (24%). At the last follow up, 7 patients (33%) had pain reduction. Median time to pain recurrence was 36 months (IQR 7-36 months). Estimated rates of meaningful pain reduction at 1, 2, 3, and 5 years were 48%, 48%, 19%, and 19%, respectively
Keep et al. [26]	2006	Retrospective	Post-stroke thalamic pain	Model B Leksell Gamma Knife unit (Elekta Instrument AB).	1	140 Gy	Centromedian nucleus of ipsilateral thalamus	83	Gradual improvement of pain, comfort and tolerance to contact leading to complete relief 12 weeks after SRS. Mild perilesional edema was observed at 12 weeks on MRI with no neurological deficit. Improvement of pain and quality of life persisted until the last follow up 6 y and 11 m after SRS.
Frighetto et al. [27]	2004	Prospective	Intractable pain (unilateral poststroke pain, oncologic pain due to metastatic infiltration of the brachial plexus)	Dedicated linear accelerator (Novalis, BrainLab, GmbH, Heimstetten, Germany)	3	150-200 Gy	Centromedian nucleus of thalamus	36	Immediate decrease of pain intensity and improvement in activities of daily living, with significant reduction of medication intake. One patient presented recurrence at 4 months and was treated with motor cortex stimulation. One poststroke pain patient presented perilesional edema, likely due to her vascular etiology.
Young et al. [28]	1994	Retrospective	Intractable pain (Structural spinal disorders, post-herpetic neuralgia, spinal injury, thalamic syndrome, anesthesia dolorosa, brainstem infarction)	Leksell Gamma Knife	10	160-180 Gy	Intralaminar nuclei of thalamus, lateral portion of the mediodorsal nucleus, and centromedian nucleus of thalamus	3-15	Median thalamotomy reduced the patients’ attention to pain with no reductions in attention or motivation. Pain relief occurred two to four weeks after SRS. Three patients presented excellent pain relief (≥50% pain reduction, no medication use and significant improvement in daily activities), four had good relief (At least 50% reduction, minimal medication use, some improvement in daily activities), and three were considered failures.
Young et al. [29]	1995	Retrospective	Intractable pain (Structural spinal disorders, post-herpetic neuralgia, spinal injury, thalamic syndrome, anesthesia dolorosa, brainstem infarction, post-amputation pain, recurrent benign sacral tumor)	Leksell Gamma Knife	19	140-180 Gy	Intralaminar nuclei of thalamus, lateral portion of the mediodorsal nucleus, and centromedian nucleus of thalamus	1-21	Four (27%) patients presented complete relief and suspended medications, five (33%) experienced improvement greater than 50% and reduced medication use, and six had minimal to no improvement. Distinct lesions started developing after 3-6 weeks and were fully formed by 8-12 weeks. Medial thalamotomy had a complication rate of 6%, with one patient developing transient progressive headache, diplopia, vomiting and contralateral upper limb weakness. This patient was treated using two isocenters and a 160 dose, and presented an unexplained increase in lesion volume that later decreased.
Steiner et al. [30]	1980	Retrospective	Intractable oncologic pain	Leksell Gamma Knife	52	10-25 krad (100-250 Gy)	Bilateral or unilateral ventromedial thalamic nuclei, CM-Pf complex; ipsilateral to the side of pain	1-105	Good to moderate relief was achieved in 13 patients treated with unilateral lesions and 14 with bilateral lesions, and slight or no results were obtained in 12 patients with unilateral lesions and 12 with bilateral lesions. Of the 8 patients with good response, pain recurred in 3 after 3-8 months. No lesions were visible with doses under 14 krad (140 Gy) and were consistently developed with lesions over 160 krad (160 Gy). Significant relief developed immediately in two-thirds of patients and persisted for 2-4 days, after which it had a tendency to reappear until final response settled in 2-4 weeks. This early relief merged with the final response in 30% of cases. Better results were obtained when pain was located in the upper body.

For patients in the current study treated with dual-target SRS, the median time to pain relief was significantly shorter, at 2.3 days, compared to the longer duration required for single-target thalamotomy. While relief was more sustained throughout the year in patients who experienced a substantial, quick relief of pain, many patients encountered rebound pain episodes after initial relief until the thalamic lesion matured, typically taking three or more months.

Patient selection and safety

Careful patient selection and long-term follow-up are essential and should be managed by a multidisciplinary team experienced in trigeminal neuralgia and facial neuropathic pain. Ideally, this team would include specialists in pain management (algology), neuropsychiatry, maxillofacial surgery, and neurosurgery.

Dual-target radiosurgery to the medial thalamus is considered safe. The most common side effect was mild paresthesia, likely due to nerve irradiation, reported in 10.3% (n=6, 10.3%) of patients. Other transient side effects included alopecia (n=1, 1.72%), headache (n=1, 1.72%), or a combination of paresthesia and one of these symptoms (n=3, 5.17%). One patient developed delayed thalamic radionecrosis and trigeminal neuritis eight months after treatment with the CyberKnife system, requiring medical management. Our findings suggest that CyberKnife has a less favorable dose profile compared to Gamma Knife technology [23]. No side effects were reported in patients treated with single or bilateral thalamotomy in this study.

Limitations

This study has several limitations. As a retrospective analysis, it is subject to potential biases in patient selection and data collection, which may affect the generalizability of the findings. The relatively small sample size, particularly for certain subgroups, limits the ability to draw definitive conclusions about the efficacy of specific radiosurgical regimens for different types of pain. Additionally, follow-up duration varied among patients, with some having limited long-term data, which may influence the accuracy of pain relief and recurrence assessments. The study also lacks a control group, such as patients receiving sham treatment or alternative therapies, making it difficult to establish causality between the radiosurgical interventions and observed outcomes. Lastly, the absence of standardized criteria for defining a neuromodulatory effect of radiation, and the variability in treatment protocols across different technologies may contribute to inconsistent results. Further prospective studies with larger cohorts and standardized methodologies are needed to confirm these findings and establish optimal treatment parameters.

## Conclusions

Stereotactic medial thalamotomy is an effective strategy for managing chronic intractable pain, providing durable relief for both malignant and non-malignant conditions. Multi-target radiosurgery may play a future role in treatment strategies for functional pain disorders. However, further research is needed to validate the use of different anatomical targets, radiation doses, and volumes. Based on our experience, multi-target radiosurgery appears safe, with no significant morbidity observed, though longer follow-up is necessary to confirm these findings. The potential of multi-target radiosurgical strategies to address the complex dimensions of chronic pain could improve patients' quality of life. Continued follow-up will be crucial to understand the long-term outcomes, including pain relief and recurrence.
