# Correction: Radiosurgery to the Medial Thalamus for Chronic Pain: A Single Group Experience and Review of Literature

**DOI:** 10.7759/cureus.c231

**Published:** 2025-07-11

**Authors:** Paola Del Cid, Alejandra Moreira, Fidel Campos, Claudia Cruz, Alejandro Blanco, Eduardo E Lovo

**Affiliations:** 1 Medicine, Dr. José Matías Delgado University, San Salvador, SLV; 2 Neurosurgery, International Cancer Center, San Salvador, SLV; 3 Radiosurgery, International Cancer Center, Diagnostic Hospital, San Salvador, SLV; 4 Anesthesia and Pain Management, Diagnostic Hospital, San Salvador, SLV; 5 Radiosurgery, Robotic Radiosurgery Center, San Jose, CRI; 6 Neurosurgery-Gamma Knife Program, International Cancer Center, Diagnostic Hospital, San Salvador, SLV

This article has been corrected to reflect that The Centro Internacional de Cancer, Hospital de Diagnostico Ethics Committee solely approved the study and issued approval number CECIC 2024/001.

